# Efficacy, safety and methodological quality of light therapy and sleep improvement interventions for people with attention deficit hyperactivity disorder (ADHD)

**DOI:** 10.1192/j.eurpsy.2023.1036

**Published:** 2023-07-19

**Authors:** D. M. Skrzypiec, B. Helfer

**Affiliations:** 1 Meta Research Centre; 2 Institute of Psychology, University of Wrocław, Wrocław, Poland

## Abstract

**Introduction:**

Large proportion of patients with ADHD experience sleep problems. Well conducted, good quality clinical research could identify non-pharmacological sleep improvement interventions that would benefit patients with ADHD and would inform evidence based guidelines for sleep management in ADHD.

**Objectives:**

To conduct a novel meta-research assessment of available clinical trials in the field of light therapy and non‑pharmacological sleep improvement interventions for people with ADHD.

**Methods:**

Peer-reviewed publications of clinical trials were analysed. An advanced literature search strategy was performed in major medical databases, including EMBASE, MEDLINE, the Cochrane Central Register of Controlled Trials and PsycINFO. Available data at WHO-approved clinical trial registries were searched and linked to the published literature. Detailed methodological assessment of results was conducted using the Cochrane Risk of Bias Tool version 2.0 (ROB2), conflict of interest, spin and favourability of findings. Reduction in ADHD symptom severity and improvement of sleep quality served as primary outcomes for the efficacy analysis. Any adverse events were recorded. Statistical analysis of the primary outcomes was conducted by calculating standardised mean difference and transformed as necessary. Publication bias was evaluated with contour enhanced funnel plots and the trim-and-fill procedure, and by summarising unpublished trials.

**Results:**

Analysed clinical trials often had a high risk of bias (evaluated by the ROB2). The primary outcome interpretation and overall trial conclusions frequently favoured the trial intervention. Clinical trials showed an association between primary outcome effect size and interpretation, and risk of bias. Clinical research in this field faces many of the same challenges identified for complex interventions in mental health, such as small sample size, lack of funding and difficulties with blinding.

**Conclusions:**

Clinical research regarding light therapy and non‑pharmacological sleep improvement interventions for ADHD patients indicates safety and effectiveness but studies often lack methodological rigour.

**Disclosure of Interest:**

None Declared

